# Changes in exclusive breastfeeding practices and its determinants in India, 1992–2006: analysis of national survey data

**DOI:** 10.1186/s13006-015-0059-0

**Published:** 2015-12-29

**Authors:** Nomita Chandhiok, Kh. Jitenkumar Singh, Damodar Sahu, Lucky Singh, Arvind Pandey

**Affiliations:** Indian Council of Medical Research, Ansari Nagar, New Delhi, 110029 India; National Institute of Medical Statistics, Ansari Nagar, New Delhi, 110029 India

**Keywords:** Exclusive breastfeeding, Determinants, Changes, Trends, India

## Abstract

**Background:**

Exclusive breastfeeding up to six months is considered to be beneficial for the health and wellbeing of infants and mothers. To guide policy makers in the development of targeted breastfeeding promotion strategies, changes in the effect of predictor variables on exclusive breastfeeding practices in India were examined.

**Methods:**

Data from two rounds of the National Family Health Survey (NFHS) carried out in India during 1992–93 (NFHS-1), and 2005–06 (NFHS-3) were analysed. A total of 34,176 and 25,459 births under three years of age in NFHS-1 and NFHS-3 respectively comprised the sample. Exclusive breastfeeding was defined as infants zero to five months of age who received only breast milk in previous 24 h. The practice of exclusive breastfeeding was examined at different ages (1, 4 and 6 months) against a set of predictor variables using bivariate and multinomial logistic regression in conjunction with the multiple classification analysis.

**Results:**

Overall 46.3 per cent and 48.6 per cent of infants under six months of age were exclusively breastfed in NFHS-1 and NFHS-3 respectively. The proportion declined with each additional month of age, and at four months only 24 per cent infants in NFHS-1 and 31 per cent infants in NFHS-3 were exclusively breastfeeding. In the NFHS-1 a higher proportion of infants perceived to be small size at birth and those with mothers in gainful employment were exclusively breastfed. While in infants of mothers living in urban areas, older mothers (aged ≥ 35 years), more literate mothers, belonging to a higher standard of living index, preceding birth interval less than two years, and in those who had antenatal/natal care, a lower proportion of exclusive breastfeeding was observed at different ages of the infant. However, in the NFHS-3, children of older mothers and of those who were less educated the proportion of exclusive breastfeeding was significantly greater at one month of age. In the age segment one to four months; exclusive breastfeeding was significantly lower in infants born to older mothers, from medium standard of living households and perceived to be of small size at birth. Infants of mothers who were more educated, aged ≥ 35 years, living in urban areas and who had antenatal/natal care were the factors associated with a lower proportion of exclusive breastfeeding at six months of age.

**Conclusions:**

The rate of exclusive breastfeeding in India continues to be sub-optimal with no appreciable gains in the last ten to fifteen years. Interventions that seek to increase exclusive breastfeeding should be timely with an increased focus on mothers with infants four to six months of age and in those who are most at risk of early discontinuation of exclusive breastfeeding.

## Background

The World Health Organization (WHO) recommends exclusive breastfeeding (EBF) to six months of age [1, 2]. During this six month period, no other liquid, semi solid or solid food or breastfeeding substitute should be given to the infants except for medicine and/or oral rehydration solution. EBF is beneficial to the health and wellbeing of infants and mothers [3]. Children who are not breastfed exclusively for six months have a higher risk of gastrointestinal infections, respiratory illness, morbidity and death [4–6], as well as atopic eczema [5, 7], allergy, asthma, type II diabetes [8], leukemia [9] and obesity in later life [3] than EBF infants. EBF is estimated to prevent approximately one-tenth of child deaths and could play an important role in meeting India’s Millennium Development Goal 4 of reducing child mortality. Cognizant of the high prevalence of inappropriate child feeding practices and the importance of exclusive breastfeeding, the Indian Government developed the Infant and Young Child Feeding (IYCF) guideline in 2004 [10], and improving infant and young child feeding practices with a strong emphasis on promotion of exclusive breastfeeding for 6 months, is a priority under the governments various flagship programs such as Integrated Child Development Services (ICDS) and the National Rural Health Mission (NRHM). Since then, varying levels of interventions, giving due emphasis to key messages of exclusive breastfeeding, are being given both at health institution and community level.

Despite the numerous recognized advantages of appropriate feeding practices, the rates of EBF in India continue to be low [11]. The success of a nutrition program depends on identification of modifiable factors susceptible to intervention. Importantly, studying inhibiting risk factors that determine the duration of EBF would serve as the basis for designing and implementing effective programs targeting individuals, families and communities at increased risk for suboptimal feeding behaviors. In addition, it would evaluate the success of ongoing programs and of various other activities promoting breastfeeding.

A number of international studies have identified several socio-demographic determinants of EBF. Some of the most common factors found to be associated with EBF are the economic status of family; education of mother; occupation of mother; utilization of antenatal care services; place of residence and access to information [12–15]. While studies assessing factors associated with continuation of breastfeeding during infancy and beyond have been conducted at the national level in developing countries like Timor-Leste [16], Bangladesh [17, 18], and Nepal [19], there are very few studies at the national level from India exploring changes that may have occurred in EBF practices evaluated at two time points when the National Family Health Survey (NFHS) in 1992–93 (NFHS-1) and 2005–06 (NFHS-3) were carried out. The NFHS are equivalent to the Demographic and Health Surveys in most other countries. This paper examines changes in EBF between the two large scale surveys and the effect of predictor variables in the context of the WHO and Government of India feeding guidelines and recommendations [1, 10].

## Methods

### Data

This study uses data from two rounds of the National Family Health Survey (NFHS) conducted in 1992–93 (NFHS-1) and 2005–06 (NFHS-3). Data from the NFHS-2 were not considered in the analysis as significant changes in EBF practices were not expected to have occurred in the five to six years between successive surveys.

The NFHS is a nationally representative cross-sectional study using a multistage cluster sampling design with internationally validated instruments. The survey is conducted every five years under the stewardship of the Ministry of Health and Family Welfare, Government of India and coordinated by the International Institute of Population Sciences, Mumbai. The key aim of NFHS is to provide national level data on key indicators like fertility, family planning, infant and child morbidity and mortality, maternal and reproductive health and nutritional status of mothers and infants.

In each of the NFHS, all currently married eligible women were asked to provide information on complete birth history including sex, month and year of birth and survival status for each live birth. Detailed information was obtained on antenatal and delivery care, breastfeeding duration and practices, vaccination and recent illnesses for the recent births which had occurred for each eligible woman during the four years preceding the survey in NFHS-1and five years preceding the survey in NFHS-3. The area covered by each of the surveys accounted for 99 per cent of the country’s population and the survey response rate ranged between 89–100 per cent across different states.

Sampling design, sample size and response rate details are published in the round-specific survey reports [20, 21]. All study procedures, consent forms and tools were approved by the Ethics Review Board of the International Institute for Population Sciences, Mumbai, India (IIPS 1992–93, IIPS 2005–06).

For the purpose of the present study, only 19 bigger states (18 states and the National Capital region of Delhi) excluding the six north-eastern states were considered for the final analysis. In these 19 States, a total of 83,511 and 102,572 eligible women respectively in NFHS-1 and NFHS-3 were interviewed. Our window of observation for analysis in this research is three years of life in both the surveys, i.e., NFHS-1 and NFHS-3, based on retrospective data of children who were aged 0–35 months at the time of survey. The analysis would therefore refer approximately to the period 1990–1993 for NFHS-1 and 2003–2006 for NFHS-3.

The aforesaid 19 states of India were further grouped into high, moderate and low post-neonatal mortality regions in view of their association with the socio-economic differentials and breastfeeding practices. Sample Registration System (SRS) figures on post neonatal mortality rate (PNMR) for the period 1990–92 [22] were used to group the states/provinces in three groups: states with high level of PNMR ≥ 27/1000 live births (Odisha, Uttar Pradesh, Bihar, Assam, Madhya Pradesh, Rajasthan, Haryana); states with medium level of PNMR ≥ 20/1000 live births (West Bengal, Andhra Pradesh, Gujarat, Karnataka, Punjab, New Delhi) and states with low level of PNMR < 19/1000 live births(Tamil Nadu, Himachal Pradesh, Maharashtra, Jammu, Goa, Kerala).

### Study variable

In the present study we used exclusive breastfeeding (EBF) among infants aged < 6 months as the outcome variable. This was defined, based on the WHO key infant feeding indicators and the guide to DHS statistics [23, 24] as the infants 0–5 months of age who received only breastmilk in the previous 24 h. From NFHS-1, information on duration of exclusive breastfeeding of currently breastfeeding children and of those who had died was included while data of only currently breastfeeding children was included from NFHS-3. The status of exclusive breastfeeding was ascertained at each month. Since the ages when the child started receiving plain water and other liquid and solid foods were recorded in completed months, for analysis purposes, we have added 0.5 months in computation of present age, or ages when the child started receiving water or other food supplements.

### Independent variables

The independent variables included the demographic and socio-economic characteristics of mothers and antenatal and natal care. In order to see the effect of mother’s age at the time of child birth on her breastfeeding practice, it was categorised as woman under 20 years, 20 – 34 years, or 35 years or more. Mother’s place of residence was categorized into rural and urban. For the work status of mother, those engaged in household work or working without wages, were categorized as non-gainful and the rest were considered in gainful occupation. The economic status of the household was measured by a composite index called standard of living index (SLI) by summing up the scores based on different facilities and items present in the household such as type of the house, source of lighting and ownership of various consumer durables [25] and were categorized into three groups as low, medium and high SLI.

To study the variation in the exclusive breastfeeding pattern in the context of educational attainment of mother, we considered three categories: (1) illiteratemothers and those who do not have any formal schooling, (2) mothers with 1–7 years of schooling and (3) mothers with 8 or more years of schooling. This grouping corresponds to the educational levels as illiterate, primary and middle school, and high school and above respectively. Thelength of the preceding birth interval was consideredintwocategoriesandwas child of first birth order or preceding birth interval < 24 months and the other if the preceding birth interval ≥ 24 months. The effect of the size of child at birth as perceived by mother was also considered under the two categories of large/average and small.

In the absence of direct information on use of health care services, proxy measures such as number of antenatal appointments, number of tetanus toxoid injections (TT) received by mother and place of delivery were used as indicators of access to use of health care facilities. A ‘variable antenatal and natal care’ was created. If mother received at least three antenatal appointments or was vaccinated against tetanus (received two doses of TT immunization) during pregnancy or delivered the child in a health facility then antenatal and natal care received by the mother was coded as ‘Yes’, otherwise, coded as ‘No’.

In sequence of above independent variables, children whose mothers were in the age group 20–34 years at last childbirth, from rural area, illiterate, with non-gainful occupation, from low SLI household, having first birth/preceding birth interval ≥ 2 years, not availing antenatal or natal care and having large/average size of the baby, were considered as reference categories of predictor variables.

### Statistical model

The rate of EBF was reported at age one, four and six months to identify changes that may occur according to the age of the infant during the surveys. This was examined against various aforesaid demographic, socioeconomic and service related characterizes of the mothers using univariate and bivariate analysis. As the study variable (type of breastfeeding: exclusively breastfeeding, breastfeeding and water only, breastfeeding with supplements and not breastfeeding) had more than two categories, the associations between exclusive breastfeeding and the aforesaid predictors (those found significant in bivariate analysis) were examined using multinomial logistic regression model controlling for potential confounders [26, 27].

Utilizing the data on the EBF status of children at different ages of the infant in NFHS-1 and at the time of survey in NFHS-3 the effects of predictor variables on EBF at the beginning of 1, 4, 6 months were estimated separately. For each variable, the effects of the other variables included in the model were controlled by setting them at their mean values. Three multinomial logistic regressions in conjunction with multiple classification analysis were estimated, one for each of two groups of states (high and medium/low PNMR) and one for all states combined. This analysis accounted for study design and sample weight as the raw data from NFHS provides sampling weights. The statistical analyses were conducted using Stata Version 13 with SVY commands that adjusted for the complex cluster sampling design used in the survey. All tests were two-sided and *p* < 0.05 was considered statistically significant. An unweighted total of 34,176 and 25,459 births under three years of age in the NFHS-1 and NFHS-3 respectively were assessed for the WHO defined EBF indicator and there were 2278 and 1896 mother-infant pairs (less than six months) in the 1992 and 2005 NFHS survey respectively.

## Results

Table 1 presents the socio-demographic profile of children born four years and five years preceding NFHS-1 and NFHS-3 respectively. In the sample of NFHS-1, there were 51 per cent male and 49 per cent female children against 52 per cent male and 48 per cent female children in NFHS-3. In both the surveys, majority of children belonged to rural areas and were born to mothers in the prime reproductive age group of 20–34 years. Two-third (66.7%) of children in NFHS-1 and half (49.7%) in NFHS-3 were from mothers who had not received any formal education. More children born to mothers in NFHS-3 were gainfully occupied (35.6 % vs 13.3 %). Nearly two-fifths of children were born to mothers with a low SLI (39.9 %) in NFHS-1 as compared to 30.1 % in NFHS-3.

**Table 1:** Characteristics of children born during the four years and five years preceding the survey in states^b^ with high and medium/low levels of post neonatal mortality, NFHS-1 and NFHS-3 respectively

Characteristics		States with level of PNMR^a^					
		NFHS-1			NFHS-3		
		High	Medium/low	All	High	Medium/low	All
Sex of the child	Male	51.3	50.8	51.1	51.8	52.6	52.1
	Female	48.7	49.2	48.9	48.2	47.4	47.9
Size at birth							
	Large/average	77.7	77.1	77.4	76.8	77.5	77.1
	Small	20.3	22.2	21.1	23.2	22.5	22.9
	Not available	2.1	0.7	1.4			
Mother’s age at birth							
	Less than 20 years	20.8	26.5	23.3	19.4	22.0	20.5
	20 - 34 years	72.2	70.5	71.4	75.1	75.7	75.3
	35 years or more	7.0	3.0	5.2	5.5	2.2	4.2
Birth order							
	1	24.4	31.8	27.7	25.7	37.4	30.4
	2 -- 3	38.3	45.7	41.6	39.9	49.6	43.8
	4 or more	37.2	22.6	30.7	34.4	13.1	25.8
Interval since last birth							
	First birth/preceding birth interval 2 years or more	81.3	82.2	81.7	72.5	74.1	73.1
	Preceding birth interval less than 2 years	18.7	17.8	18.3	27.5	25.9	26.9
Antenatal & natal care							
	Yes	42.7	81.7	59.9	61.3	86.8	71.6
	No	57.3	18.3	40.1	38.7	13.2	28.4
Residence							
	Urban	16.2	31.1	22.7	18.2	36.0	25.4
	Rural	83.9	68.9	77.3	81.8	64.0	74.6
Mother’s education							
	Illiterate	76.9	53.8	66.7	61.9	31.6	49.7
	Literate, < middle school complete	11.5	22.6	16.4	17.3	27.9	21.6
	Middle school complete & above	11.6	23.6	16.9	20.8	40.5	28.7
Mother’s occupation							
	Non gainful occupation	89.5	77.7	84.3	62.2	67.8	64.4
	Working for someone else	8.4	19.6	13.3	37.8	32.2	35.6
	Self employed	2.2	2.6	2.4			
Caste							
	Scheduled caste (SC)	10.8	6.7	9.0	21.6	21.0	21.4
	Scheduled tribe (ST)	14.7	12.0	13.5	10.1	8.1	9.3
	Other than SC/ST	74.5	81.3	77.5	68.3	70.9	69.3
Standard of living index							
	Low	41.0	38.5	39.9	35.0	22.8	30.1
	Medium	46.8	45.9	46.4	32.2	30.4	31.5
	High	9.7	14.1	11.7	22.7	37.5	28.7
	Not available	2.4	1.5	2.0	10.1	9,3	9.7

^a^ *PNMR* Post Neonatal Mortality Rate

^b^North eastern states are excluded

In the NFHS-1, 27.7 per cent children were first order births, 18.3 per cent were born with birth spacing of < 2 years and more than half (59.9 %) were to mothers who had accessed antenatal/natal care. One in every five (21.1 %) infants was perceived by the mothers to be smaller than large/average size at birth. In the NFHS-3, a nearly equal proportion (30.4 %) were first order births, 26.9 per cent were with birth spacing < 2 years and around three-quarters (71.6 %) of children were born to mothers who had received antenatal/natal care. The proportion of infants who were perceived by the mothers as small at birth was 22.9 per cent, similar to what was found in NFHS-1.

Table 1 depicts that in both the surveys, the children with more advantage in terms of their mother being more educated, belonging to high standard of living, urban residence or use of health services like antenatal and natal care, belonged to medium/low post neonatal mortality rate (PNMR) states.

### Trends in exclusive breastfeeding

Exclusive breastfeeding among children 0–6 months of age was widely practiced in most states in the first month of life. However, EBF declined with each additional month and by the time infants are 6 months of age, exclusive breastfeeding rates were low. In states where PNMR was high, 52 per cent of children at one month, about one-quarter of children at four months and one-seventh (14 %) at six months received exclusive breastfeeding in NFHS-1. The corresponding estimates for NFHS-3 were greater with 65 per cent of children beyond one month and over 30 per cent beyond four months were exclusively breastfed. At six months, this estimate was the same at 14 per cent in both the surveys, i.e., NFHS-1 and NFHS-3. A similar increase in the exclusive breastfeeding rates between the two surveys was apparent in states with medium/low PNMR at 1, 4, 6 months (Table 2).

**Table 2:** Life table estimates of percentages of women exclusive breastfeeding by age of child and type of PNMR^a^ state

	NFHS-1			NFHS-3		
At exact age (in month)	High PNMR state	Medium/low PNMR state	Any PNMR state	High PNMR state	Medium/low PNMR state	Any PNMR state
1	52	52	52	61	69	65
2	41	43	42	48	59	52
3	31	35	33	41	51	45
4	24	23	24	29	34	31
5	19	19	19	20	24	21
6	15	13	14	12	16	14
7	8	7	7	12	8	11
8	7	5	6	3	7	5
9	5	4	4	3	4	3
10	4	3	3	3	2	3
11	4	2	3	1	3	2
12	3	2	3	0	2	1

^a^ *PNMR* Post Neonatal Mortality Rates

Table 3 presents the state-wise proportion of children exclusively breastfed less than six months of age for both the surveys. For children less than one month of age in NFHS-1, the proportion of children being exclusively breastfed ranged from a low of 6.3 per cent in Punjab to 80.3 per cent in Assam. Over three-quarter of children were exclusively breastfed for at least one month only in the states of Andhra Pradesh, Assam, Kerala and Rajasthan. Less than half of children were exclusively breastfed in 9 out of the 19 states. The proportion of babies exclusively breastfed subsequently shows a gradual decline, and for infants aged 4–5 months around two-fifths are exclusively breastfed only in Assam, Karnataka, Rajasthan and Uttar Pradesh. Overall, 46.3 per cent of infants aged 0–5 months were exclusively breastfed and, except in the states of Punjab, Goa and Jammu region where it was very low (<10 %), one-quarter to half the babies in the other states receive exclusive breastfeeding. There was no appreciable difference between the high, medium and low PNMR states.

**Table 3:** State-wise proportion of children exclusively breastfed according to child’s age in months for living children less than 6 months of age in NFHS-1 (1992-93) and NFHS-3 (2005-06)

Name of State	% Exclusive breastfeeding in NFHS-1								% Exclusive breastfeeding in NFHS-3							
	0-1 months		2-3 months		4-5 months		0-5 months		0-1 months		2-3 months		4-5 months		0-5 months	
	n	%	n	%	n	%	n	%	n	%	n	%	n	%	n	%
High post neonatal mortality states																
Assam	62	80.3	55	53.4	36	43.4	153	58.2	43	84.7	40	62.1	28	46.1	111	63.1
Uttar Pradesh	237	68	248	54.8	163	40.1	648	53.6	147	70.7	172	53.9	102	29.7	422	48.5
Madhya Pradesh	45	38.4	40	26	28	16.9	113	25.9	61	57.5	74	43.8	67	37.9	203	44.9
Haryana	24	46.1	32	32.9	19	20.4	74	31		*	7	21.2	1	1.9	17	16.9
Rajasthan	76	78.4	88	57.9	50	39.7	214	57.1	29	62.1	29	34.5	15	16.9	73	33.2
Orissa	33	59.4	31	36.6	20	21.1	84	35.6	36	79	28	47.9	25	35.3	88	50.8
Bihar	55	57.1	88	48.9	57	34.2	199	45.1	62	63.9	40	43	31	21.4	133	39.7
Medium post neonatal mortality states																
Andhra Pradesh	51	78.5	40	62.5	32	33	123	54.4	28	79.1	57	77.3	24	37.3	109	62.7
West Bengal	26	43.6	21	36.2	17	19	64	30.8	63	78.8	64	64.9	23	29.3	150	58.6
Karnataka	51	69.9	71	62.8	40	43	162	58.1	38	81	34	61.1	28	41.3	101	58.6
Gujarat	26	45.6	27	30.3	14	15.7	67	28.5	30	75	23	50	12	24	65	47.8
Punjab	4	6.3	0	0	0	0	4	2.3	19	54.7	18	31.8	10	24.9	47	35.7
Delhi	13	27.7	11	15.1	5	6.1	29	14.3		*	15	27.6	14	29.5	42	34.5
Low post neonatal mortality states																
Tamil Nadu	29	64.4	34	50	12	21.8	75	44.6	21	59.7	16	39.7	14	19.2	51	34.1
Kerala	31	75.6	40	50.6	10	14.5	81	42.8		*	19	59.3	11	39.3	41	56.2
Himachal Pradesh	31	45.8	15	25.7	11	17.5	58	30.1		*	12	27	3	7.5	27	27.1
Goa	8	10.8	5	5.5	2	1.7	15	5.3		*	8	25.2	1	3.6	14	17.7
Maharashtra	34	46.6	32	30.5	28	26.7	94	33.2	52	70.2	75	61.9	35	31.8	162	53
Jammu Region	15	30.7	5	7.2	1	1.7	21	11		*	22	55.2	9	27.8	40	42.3
Total (19 states)	851	63.0	883	48.7	545	33.1	2278	46.3	629	70.9	753	53.3	453	31.8	1896	48.6

*percentage not shown; based on fewer than 25 unweighted cases

NFHS-3 data indicated an increase in exclusive breastfeeding in all the states for infants less than one month of age except in Rajasthan in the high PNMR states, where there was a decline from 78 per cent to 62 per cent. In 7 out of the 19 states, more than three-quarters of babies aged 0–1 month were exclusively breastfed and of these, four states were in medium PNMR states. At age 2–3 months, more than 50 per cent infants in 9 states were exclusively breastfed. Exclusive breastfeeding reduced as the child grew older. At age 4–5 months, surprisingly in 7 states, a higher percentage of exclusive breastfeeding was observed in NFHS-1. Overall, 48.6 per cent of mothers with infants less than six months practiced EBF.

### Effect of predictor variables on EBF

Tables 4, 5 and 6 give the effect of predictor variables at the exact age of child 1, 4 and 6 months.

**Table 4:** Adjusted percentage of children who were on exclusive breastfeeding at the age of one month by socioeconomic category of mother and household during NFHS-1 & NFHS-3

Characteristics	Reference category	NFHS-1						NFHS-3					
		States with level of post neonatal mortality											
		High		Medium/low		Any		High		Medium/low		Any	
		Adjusted percentage of women practicing each type of breastfeeding											
		EBF	NBF	EBF	NBF	EBF	NBF	EBF	NBF	EBF	NBF	EBF	NBF
Residence	Urban	43*	2	54*	2	50*	2	57**	9	72	6	65	6
	Rural^+^	56	1	61	1	58	1	63	7	68	4	65	6
Mother’s age at birth (years)	<20	54	1	58	2	56	1	56	11	69*	4	62	7
	20 – 34^+^	56	1	61	1	59	1	61	7	68	5	64	6
	35 & above	50**	2	53*	3	51*	2	81**	0	96**	0	85**	0
Mother’s education	Illiterate^+^	56	1	63	1	58	1	59	7	72	4	64	6
	Literate, < middle school complete	50	1	52*	2	51*	1	61	12	73**	5	67	8
	Middle school complete & above	47	2	56*	2	52*	2	63	6	66	5	64	5
Mother’s occupation	Non gainful occupation+	54	1	57	2	55	1	59	8	64	5	61	7
	Gainful occupation	59	1	65	2	64	1	65	7	83*	2	73	5
Standard of living index	Low^+^	56	1	63	2	60	1	61	8	81	0	68	5
	Medium	54	1	58	2	56	1	65	6	67**	6	67	6
	High	47	1	48	2	46*	2	58	8	64*	7	61	7
Interval since last birth	First birth/preceding birth interval 2 years or more^+^	55	1	59	2	56	1	67	2	74	4	70	3
	Preceding birth interval less than 2 years	50*	2	58	2	54**	2	47**	18	54	4	51	13
Antenatal & natal care	Yes	49*	1	59	2	55*	2	62	8	69	5	66	6
	No^+^	58	1	56	2	57	1	58	7	72	1	61	6
Size of the baby	Large/average^+^	53	1	58	1	55	1	62	5	70	4	66	4
	Small	58*	2	60*	3	59*	2	58	16	67	7	63	12
All		54	1	58	2	56	2	61	7	69	5	65	6

Note: 1. *EBF* exclusive breastfeeding, *NBF* not breastfeeding 2. The percentages shown are adjusted by multiple classification analysis in conjunction with multinomial logistic regression. A plus (+) indicates a reference category and asterisk (*) or asterisks (**) indicate that the underlying multinomial logistic regression coefficient, associated with ratios of probability of exclusive breastfeeding (EBF) to the probability of no breastfeeding (NBF) differences significantly from zero at the 5 % or 1 % level respectively

**Table 5:** Adjusted percentage of children who were on exclusive breastfeeding at the age of four month by socioeconomic category of mother and household during NFHS-1 & NFHS-3

Characteristics	Reference category	NFHS-1						NFHS-3					
		States with level of post neonatal mortality											
		High		Medium/low		Any		High		Medium/low		Any	
		Adjusted percentage of women practicing each type of breastfeeding											
		EBF	NBF	EBF	NBF	EBF	NBF	EBF	NBF	EBF	NBF	EBF	NBF
Residence	Urban	14*	4	24*	6	21*	5	15	6	23	12	19	6
	Rural^+^	24	2	29	4	27	3	32	6	39	9	35	5
Mother’s age at birth (years)	<20	22	3	26*	4	24**	3	28	6	29	12	29	7
	20 – 34^+^	24	3	32	4	29	3	24	5	35	8	28	5
	35 & above	22	3	23	6	23	3	41	8	3**	49	38	11
Mother’s education	Illiterate^+^	24	2	34	4	28	3	31	6	40	20	34	8
	Literate, < middle school complete	19*	3	22*	4	21*	4	19	5	36	2	27	3
	Middle school complete & above	14*	6	22*	6	19*	5	23	5	23	4	25	5
Mother’s occupation	Non gainful occupation^+^	22	3	26	4	24	3	23	5	31	6	26	4
	Gainful occupation	26	3	33**	4	32*	3	32	7	38	21	35*	9
Standard of living index	Low^+^	25	2	35	4	30	3	29	4	48	11	36	4
	Medium	22*	3	27**	4	25*	3	32	9	23*	12	29*	8
	High	16*	4	15*	6	15*	5	18	4	29	10	24	5
Interval since last birth	First birth/preceding birth interval 2 years or more^+^	23	3	28	4	26	3	28	5	34	14	30	5
	Preceding birth interval less than 2 years	19*	3	28	4	23*	4	26	5	39	2	31	4
Antenatal & natal care	Yes	19*	3	28**	4	25*	4	21	5	32	7	26	5
	No^+^	26	3	26	3	25	3	42	5	35	22	42	7
Size of the baby	Large/average^+^	22	2	27	4	25	3	24	5	36	12	29	6
	Small	24*	3	30*	6	27*	4	32	7	23*	6	29	6
All		54	24	3	29	5	26	26	5	33	10	29	6

Note: 1. EBF: exclusive breastfeeding; NBF: not breastfeeding. 2. The percentages shown are adjusted by multiple classification analysis in conjunction with multinomial logistic regression. A plus (+) indicates a reference category and asterisk (*) or asterisks (**) indicate that the underlying multinomial logistic regression coefficient, associated with ratios of probability of exclusive breastfeeding (EBF) to the probability of no breastfeeding (NBF) differences significantly from zero at the 5 % or 1 % level respectively

**Table 6:** Adjusted percentage of children who were on exclusive breastfeeding at the age of sixmonth by socioeconomic category of mother and household during NFHS-1 & NFHS-3

Characteristics	Reference category	NFHS-1						NFHS-3					
		States with level of post neonatal mortality											
		High		Medium/low		Any		High		Medium/low		Any	
		Adjusted percentage of women practicing each type of breastfeeding											
		EBF	NBF	EBF	NBF	EBF	NBF	EBF	NBF	EBF	NBF	EBF	NBF
Residence	Urban	8*	6	13*	8	11*	7	5**	10	7*	18	5**	15
	Rural^+^	13	3	16	5	15	4	12	6	26	7	17	6
Mother’s age at birth (in years)	<20	12	4	14*	6	13*	5	12	8	24	18	16	13
	20 – 34^+^	13	4	18	6	17	5	9	7	18	9	12	9
	35 & above	12	3	12	6	12	4	5	19	0*	15	4**	16
Mother’s education	Illiterate^+^	14	3	20	5	16	4	15	6	22	12	16	8
	Literate, < middle school complete	9*	4	12*	6	11*	5	10	7	20	4	13	7
	Middle school complete & above	8*	7	11*	8	10*	7	3**	9	14	13	8	13
Mother’s occupation	Non gainful occupation+	12	4	15	6	13	5	10	7	19	10	13	9
	Gainful occupation	15	4	18	6	18*	5	8	8	17	13	11	11
Standard of living index	Low^+^	15	3	21	5	19	4	13	7	23	9	15	8
	Medium	11*	4	14	6	13*	4	10	8	21	8	14	9
	High	9*	6	7	10	8*	8	5	8	10	12	7	11
Interval since last birth	First birth/preceding birth interval 2 years or more^+^	13	4	15	6	14	4	10	4	16	9	12	6
	Preceding birth interval less than 2 years	10*	4	15	6	13*	5	8	9	30	3	15	8
Antenatal & natal care	Yes	10*	4	15*	6	14*	5	8*	6	15	13	12	9
	No^+^	15	3	16	5	15	4	13	12	35	3	16	11
Size of the baby	Large/average^+^	12	3	15	6	14	4	10	7	18	9	12	9
	Small	13*	5	16*	8	15*	6	9	10	22	18	13	13
All		14	4	17	7	15	6	12	8	16	11	14	8

Note: 1. *EBF* exclusive breastfeeding, NBF not breastfeeding. 2. The percentages shown are adjusted by multiple classification analysis in conjunction with multinomial logistic regression. A plus (+) indicates a reference category and asterisk (*) or asterisks (**) indicate that the underlying multinomial logistic regression coefficient, associated with ratios of probability of exclusive breastfeeding (EBF) to the probability of no breastfeeding (NBF) differences significantly from zero at the 5 % or 1 % level respectively

#### Effect of predictor variables on EBF at one month of age

Analysis of NFHS-1 data indicated that the proportion of infants exclusively breastfed was significantly greater in rural areas (58 %) than those from urban place of residence (50 %). Such association existed across the country. In states with high PNMR, the proportion of children exclusively breastfed was 56 per cent in rural areas and 43 per cent in urban areas. In medium or low PNMR states, 61 per cent children in rural areas were exclusively breastfed in contrast to 54 per cent in urban areas. Children of older women (35 years of age and above) were strongly associated with a decrease in exclusive breastfeeding in the high PNMR states. This association, though moderately significant, also emerged in the medium/low and any PNMR states. No effect of mother’s education was seen in the high PNMR states. However, a moderate association of EBF was present in medium/low PNMR states where children of literate mothers had a less likelihood of being exclusively breastfed. Across all states irrespective of PNMR level (high, medium and low), fewer children of mothers with high SLI as compared to low SLI were exclusively breastfed and this association was moderately significant. The exclusive breastfeeding was significantly shorter when preceding birth interval < 2 years. The proportion of children exclusively breastfed was significantly lower in mothers who availed antenatal/natal care (55 %) and this effect was seen in states with high PNMR and when all the states were considered together. Across all states regardless of the PNMR, a significantly higher proportion of babies perceived to be small at birth were exclusively breastfed.

In the NFHS-3 data, compared to NFHS-1, children of mothers living in rural areas (63 %) were more likely to exclusively breastfed than those of mothers residing in urban areas (57 %) in the high PNMR states. Unlike NFHS-1, a significantly greater proportion of infants of older mothers (≥35 years) were exclusively breastfed. This association was seen across the states. In contrast to what was observed in NFHS-1, infants of less educated mothers in medium/low PNMR states had a greater likelihood of EBF than those whose mothers were illiterate. More infants of mothers in gainful occupation (83 %) were exclusively breastfed in medium/low PNMR states as compared to infants of mothers in non-gainful occupation (64 %). There was a highly significant and moderately significant decrease in exclusive breastfeeding in children of mothers with medium and high SLI respectively in states with medium/low PNMR. In states with high PNMR, 47 per cent infants with shorter birth intervals were exclusively breastfed as compared to 67 % where the preceding birth interval was ≥ 2 years.

#### Effect of predictor variables on EBF at 4 months of age

A lower percentage of infants living in an urban residence as compared to rural residence were breastfed at four months of age in NFHS-1. A significant reduction in EBF at 4 month was observed in infants born to very young mothers < 20 years of age in medium/low PNMR states and all states irrespective of PNMR. On the whole, infants of literate mothers, across all states including states with high and medium/low PNMR, were less likely of being EBF. Though no association of mother’s occupation with EBF was evident in the high PNMR states, a significantly higher proportion of infants with mothers in gainful occupation (33 %) were EBF as compared to 26 per cent in non-gainful occupation in the medium/low PNMR states. This association of gainful occupation on exclusive breastfeeding was seen to persist even when all the states were considered together though the strength of the association was less. A significantly lower proportion of children with high and medium SLI were exclusively breastfed as compared to those with low SLI across all states including high and medium/low PNMR states. This association was stronger in infants with medium SLI and belonging to medium/low PNMR states. A lower percentage of EBF among children with preceding birth interval < 2 years in high and any PNMR states seen at one month of age persisted at four months.

A variable association was observed of EBF with utilization of antenatal and natal care. In children whose mothers had availed these health services in the high PNMR states, about one-fifth (19 %) were exclusively breastfed in comparison to over one-quarter (26 %) children whose mothers had not availed above services. However, in states with medium/low PNMR, the proportion of infants whose mothers availed antenatal services and exclusively breastfed was 28 per cent whereas the proportion of infants whose mother did not avail antenatal services and exclusively breastfed was 26 per cent and this difference was highly significant. Analogous to that seen at one month of age, a significant association between infants perceived as small size at birth and exclusive breastfeeding was seen across all states.

In the NFHS-3, no association was seen with many of the predictor variables including residence, mother’s education, interval since last birth, antenatal and natal care. In the medium/low PNMR states, only 3 per cent infants of older mothers (age ≥ 35 years) were exclusively breastfed as compared to 35 per cent infants with mothers in the age group of 20-34 years. This difference was highly significant. Infants of mothers belonging to medium SLI households in medium/low and any PNMR state were less likely to be exclusively breastfed. Similar observation was made with infants perceived as small size at birth (23 %) than those large/average (36 %) in size. In any PNMR state, over a third of infants (35 %) with mothers in gainful occupation were exclusively breastfed. Whereas this was only 26 per cent for infants with mothers in non-gainful occupation.

#### Effect of predictor variables on EBF at six months of age

In the NFHS-1, a significantly small proportion of infants living in urban areas across all states were EBF as compared to those in rural areas. A lower proportion of children of very young mothers (<20 years of age) were exclusively breastfed as compared to children of mothers in the prime reproductive age of 20-34 years. In medium/low PNMR states, 14 per cent infants born to very young mothers and 18 per cent born to mothers 20-34 years of age were exclusively breastfed. The corresponding figures in any PNMR state were 13 per cent and 17 per cent respectively. Education attainment of mother was inversely associated with EBF mothers across all states with a lower proportion of infants of literate mothers being exclusively breastfed. Infants with high/medium SLI in states with high PNMR or any state regardless of PNMR had a lesser likelihood of being exclusively breastfed. A significantly lower proportion of infants with preceding birth interval < 2 years in high PNMR states were exclusively breastfed. Similar to that observed at four months of age, infants of mothers availing health services like antenatal and natal care had a lesser likelihood of exclusively breastfeeding their child. The positive predictor variables for EBF were mother in gainful occupation in any state regardless of PNMR and small size of baby at birth where the association was seen across all states.

In NFHS-3, residence emerged as a strong predictor of EBF. As compared to urban, a significantly higher proportion of infants from rural areas in high PNMR states (12 % versus 5 %) and medium/low PNMR states (26 % versus 7 %) were exclusively breastfed. Only 3 % children of more literate mothers in high PNMR states were EBF in contrast to 15 % children from illiterate mothers and the difference was highly significant. The effect of occupation of mother on EBF was also observed at this age. A moderately significant increased likelihood of EBF was seen in infants with mothers in gainful occupation (35 %) in any PNMR state as compared to non-gainful occupation (26 %). Infants of mothers who had availed antenatal and natal services had a lower possibility of being EBF in high PNMR states.

## Discussion

Having evidence of the large variations in exclusive breastfeeding practices and the extent to which selected socio-demographic and obstetrical factors account for such variation could lead to more effective interventions. The current study examined changes in the rate and determinants of EBF in 19 states of India including 18 bigger states and the national capital region of Delhi based on the data collected from two nationally representative large scale demographic surveys carried out 13 years apart.

Our analysis revealed that despite breastfeeding promotion programs, EBF in India continues to be low. A marginal improvement was observed from 46.3 per cent in NFHS-1 to 48.6 per cent in NFHS-3 in the proportion of infants < 6 months old who were exclusively breastfed which was significantly lower than the WHO/UNICEF recommendation of 90 per cent. The EBF rates in India were similar to that reported by countries from the neighbouring region with Pakistan reporting 37.1 per cent [28], Bangladesh 42.5 per cent and Nepal 53.1 per cent [29].

An increase in EBF at each month of age was evident between the two surveys. However, this difference in EBF rates narrowed from four months of age onwards. Such findings were also reported earlier from India [30] and Pakistan [28]. The possible reason for this could be that mothers introduced complementary feeding for their infants under the assumption that breast milk alone might not satisfy the needs of infants as they grow older. It indicated an urgent need for improvement especially from 4-6 months when the EBF rates rapidly decline.

Our study assessed the effect of changes over time of a total of eight socio-economic, demographic and health care utilization factors on exclusive breastfeeding between the two surveys. In the NFHS-1, besides small size of the baby at birth and gainful employment of mother, for all other factors when compared to the reference category, EBF was seen to be significantly lower at different ages of the infant. At age one month, the duration of EBF was shorter in children born to older and urban educated women with a high standard of living index, birth spacing < 2 years and those who had availed antenatal health services or delivered in a health facility. At four months of age which is the critical period when EBF rates drop, instead of older mothers, very young mothers with a medium/high standard of living index had a negative association with EBF.

Over time there was a change in the determinants of EBF. In NFHS-3,a significantly lower proportion of infants living in urban areas, short preceding birth interval and belonging to higher wealth quintile as per SLI were exclusively breastfed at one month of age. At the same age, in children of older, less educated and mothers in gainful occupation, the proportion of EBF was significantly greater. In the age segment 1-4 months, EBF was significantly lower in infants born to older mothers with medium SLI and small size at birth. Mothers’ being in gainful occupation was the only factor associated with a higher proportion of infants being exclusively breastfed. As the child grew older, association of EBF was seen with fewer variables and at six months of age, children whose mothers were more educated, living in urban areas, from medium SLI household and availing antenatal/natal care were less likely to be exclusively breastfed than their counterpart while children of mothers who were in gainful occupation were likely to be breastfed longer. Chudasama et al. [30] reported parity, delivery interval more than 24 months, early maternal age, lower socio-economic status, low paternal education and occupation as a risk factor for early cessation of EBF.

As urbanization and modernization of society is believed to influence the life style, the present study tried to examine this by considering woman’s place of residence as rural or urban. In rural areas, people live together in a village and their beliefs and religious practices may have a strong influence on their behaviour and social habits. A significant association that infants in urban area as compared to rural area are less likely to be breastfed at the different ages of the infant emerged in the two surveys and is also reported from other countries. For instance, in a study carried out in Malaysia, mothers from rural area were more likely to exclusively breastfeed compared to mothers from urban area (OR = 1.16; 95 % CI 1.03, 1.89) [12].

Several studies report non-gainful occupation of mother as a predictor of exclusive breastfeeding [15, 31]. However, our analysis indicated a significant higher proportion of infants of mothers in gainful occupation were exclusively breastfed. This association was present in both the surveys and in all three age segments studied. An explanation for this could be increased knowledge and awareness of EBF through interaction with colleagues at workplace. Under the Maternity Benefit Act 1961, women in India are entitled to 12 weeks paid maternity leave and since 2008, this has increased to 180 days for government employees. However, these benefits are variable in the organized and unorganized sector and policies guaranteeing maternity entitlements to women working outside the formal sector need to be implemented [32].

Increasing household wealth level as measured by the validated SLI had an inverse association and led to a progressive increase in the likelihood of stopping exclusive breastfeeding. This effect was seen at all ages up to six months in the NFHS-1 for mothers living in high and medium PNMR states. In the later NFHS-3 carried out in 2005-06, no association with SLI was seen in the high PNMR states at all ages but high/medium SLI was associated with a lower proportion of exclusive breastfeeding in the medium/low PNMR states. This is consistent with previous reports of adverse impact of high SLI on duration of breastfeeding in developing countries [17, 29, 33–35]. This could be due to greater affordability in high SLI household to foods other than breast milk. The lack of association of SLI observed in the later survey in the high PNMR states could be due to focused efforts to promote EBF in these states.

Contrary to other studies [36], a lower proportion of infants born to mothers who received antenatal care during pregnancy or were born in a medical facility received EBF in the NFHS-1. However, this changed over time and in NFHS-3, no association between EBF and utilisation of health services was apparent. Government policies that have led to improvements in health care services and an increase in institutional delivery have contributed towards this. Antenatal and postnatal care was associated with higher EBF rates in Sri Lanka but in Nepal antenatal visits were found to have a negative influence [29]. Antenatal contact with a health professional provides an opportunity to counsel a woman to establish and continue EBF and lack of breastfeeding counselling was significantly associated with decreased rates of EBF at 4 and 6 months in rural north India [37]. Providing correct information in simple language by a well-trained care provider will prevent problems and help women to succeed in breastfeeding [37, 38].

The other change observed between the two surveys was the influence of mother’s education on EBF. Infants born to literate mothers had a less likelihood of being exclusively breastfed in NFHS-1which was not significant in NFHS-3. While infants of less literate mothers in medium/low PNMR states were more likely to be exclusively breastfed at one month of age, the reverse was seen in more literate mothers at six months of age in high PNMR states. In a study carried out in Ghana and in urban slums in India, mother’s education was associated with higher likelihood of EBF [36, 39].

The strength of this study is the use of validated questionnaire and nationally representative data set over two time points, very high survey response rates, low rates of missing and excluded data and appropriate adjustments for sampling design made in the analysis. The study limitations are the recall bias for exclusive breastfeeding data in NFHS-1, particularly for children who have died. The recommendation to collect data on breastfeeding practices only from children who are currently being breastfed [23, 24] could lead to an over or underestimation in the proportion of infants exclusively breastfeeding since some infants who were given other liquids irregularly may have moved in or out of the criteria in the 24 h before the survey [40–42]. This exposure misclassification could result in biased measures of association seen in such large scale surveys [43, 44]. In addition, the cross-sectional nature of this study prevents drawing causal inferences from the association between the determinant factors and exclusive breastfeeding.

## Conclusion

The rate of EBF in India continues to be sub-optimal with no appreciable gains in the last 13 years. Our study indicated that determinants of EBF change over time. The factors identified with non-compliance of EBF were living in urban areas, shorter birth intervals and belonging to higher wealth index. Interventions that seek to increase exclusive breastfeeding should be more focused on mothers with infant’s 4-6 months of age and in those who are most at risk of early discontinuation of breastfeeding. EBF needs to be supported through an integrated approach meeting the different needs and regions of this vast country and especially in states with high neonatal mortality rates where these practices are deficient. Several successful intervention models and strategies to promote EBF have been evaluated and there is a need to scale-up and implement the most appropriate and culturally acceptable ones to universalise optimal infant feeding practices. In addition, providing maternity entitlements to women would actualize the rights of mothers and infants to breastfeed.

## Abbreviations

NFHS: National Family Health Survey
EBF: Exclusive breastfeeding
SLI: Standard of living index
WHO: World Health Organization
PNMR: Post neonatal mortality rate.
